# Books Reviewed

**Published:** 1865-04

**Authors:** 


### BOOKS REVIEWED.

### TRANSACTIONS OF AMERICAN MEDICAL ASSOCIATION.—VOL. XV.

We have received the above volume of the Association's proceedings, making a book of some four hundred and fifty pages, in style and appearance every way commendable. It contains a record of the doings at the session of 1864, both of a business and scientific character, a prize essay on jaundice, an account of the plan of organization, and the code of ethics.

It appears that eighteen States, the Army and Navy, and the District of Columbia, were represented by four hundred and sixty-five delegates, and sixty-eight permanent members. After the appointment of a Nominating Committee, the retiring President, Dr. March, read an address which is published in the volume. The subject which the address was mainly devoted to was the oft discussed one, of medical education, though the retiring officer, after the manner of departing rulers, now and then, took occasion to indicate his choice of a successor—a nowise commendable precedent. This matter of medical education is always being agitated and never making any headway, being always, or we might say yearly, put forward with profuse promises to the ear, to be broken to the sense. After all the assertions put forward by teachers, there can be no doubt that every year additions in the way of creation are made to the profession, of badly educated young men, and we might say of young men whose capacities in some cases are not equal to anything better than bad education. To whomever it is due, and that is not an easy matter to decide, whether to the public who accept, or to the schools which endorse, there is no doubt some foundation for the statement that the education of the medical profession has not kept pace with that of the other professions. We can see where the remedy lies, but we can also see how little likely it is to be applied as long as pecuniary or professional ambition, or both, continue to influence teachers. However, nothing is lost by discussion, and it is a good sign to confess to a necessity for something better, even if the desire goes not with it.

In regard to the manner in which the committees appointed to report on various subjects discharged their duties, some idea may be formed, from the fact that of thirty-four different committees appointed, some twenty-five made no report—though it should be added that several asked a continuance and promised a report at a subsequent period. Of the reports presented and printed, several are of considerable interest. We might instance those on Pessaries, on Military Hygiene and on Pathology of Lateral Curvature of the spine. Of the first, though we may not be prepared to go the full length of the writer, we are inclined to say, that the opinions advanced are worthy of the most earnest consideration. It may be, as suggested by Dr. Peaslee, one of the Committee on Obstetrics; that pessaries aresometimes absolutely the only means left us; but yet we fully believe the time will come when they will be disused entirely. We are decidedly of the opinion that *as used now*, pessaries do more harm than good.

The prize essay on Jaundice appears to be a very careful and thorough investigation of the subject, in which all the modern methods of analysis, and the microscope are called in. This appears to be one more added to the many valuable essays which are found among the publications of the Association—taken collectively, forming a body of writings of the highest merit and value—enough to redeem the failures of the Association in many other respects.

It may be added in explanation of the failure of many important committees to report, that those called upon to examine important subjects, as for instance, the resection of bones, require much time and care; and therefore cannot be expected to report in a short time. On the other hand many committees are got up at the urging of some one who has a matter to present, often a hobby, or a personal interest or grievance, in which no one but the proposer is interested, and which even he may forget in a year. Such committees do well not to report.

In the report of the Committee on Insanity, though the matter has some importance, one can easily see that the "hobby horse is [not] forgot."

The "vexed question" of what is to be done with specialists and specialties, came up again and was set apart for a committee. Dr. White of this city, we notice, was excused from serving upon it, at his own request. Specialists must be in some manner recognized by the profession, repugnant as it now appears to be to most. There is no good reason why quacks should be allowed to be the only specialists. We hope when the committee is called upon they will not report, "no report."

Upon the whole, this volume of Transactions rather tends to convince us, that the noble object for which the Association was created, so simply and well expressed in an early resolve, viz:—"That it is expedient for the medical profession of the United States to institute a National Medical Association, for the protection of their interests, for the maintenance of their honor and respectability, for the advancement of their knowledge, and the extension of their usefulness," has not been fully realized. Its very constitution makes it, for the advancement of knowledge, inferior to smaller organizations, of men of like zeal, attainments and industry; kindred in tastes and sympathies. It is apparent that though the list of members contains the best names in the profession, there are many who have ends to gain, more purely personal than professional, and are more interested to gain some advantage or recognition, than to contribute to the advancement of science. Yet its labors have done much to create a wide spread feeling of brotherhood, to uphold professional honor, and to stimulate to a higher usefulness. Its Transactions will always prove of interest to the profession.

---

*Medical Lexicon—A Dictionary of Medical Science; containing a concise explanation of the various subjects and terms of Anatomy, Physiology, Pathology, Hygiene, Therapeutics, Pharmacology, Pharmacy, Surgery, Obstetrics, Medical Jurisprudence, and Dentistry; Notices of Climate, and of Mineral Water; formulæ for Officinal, Empirical, and Dietetic Preparations; with the Accentuation and Etymology of the Terms, and the French and other Synonymes; so as to constitute a French as well as English Medical Lexicon.* By Robley Dunglison, M. D., LL. D., *Professor of the Institute of Medicine, etc., in the Jefferson Medical College of Philadelphia. Thoroughly revised and greatly modified and augmented. Philadelphia:* Blanchard & Lea, 1865.

Dunglison's Medical Dictionary has so long been our standard work that little need be said of its merits; it deservedly stands at the head, and cannot be surpassed in excellence. The present edition has been very carefully revised and enlarged, about seventy pages having been added. Great attention and labor has been bestowed in perfecting the etymology and accentration of terms, and rendering the work so complete as to embrace every term that has been "legitimated in the nomenclature of the science." This work has now more attractions than ever for the practitioner and student of medicine.

---

*The Functions and Disorders of the Reproductive Organs in Childhood, Youth, Adult Age and Advanced Life, considered in their Physiological, Social and Moral relations.* By William Acton, M. R. C., *late Surgeon to the Islington Dispensary, and formerly Externe to the Venereal Hospitals Paris, Fellow of the Royal Med. and Chir. and Statistieal Societies, etc., etc. From the last London edition. Philadelphia:* Linday & Blakiston, 1865.

We have to beg the pardon of the publishers of this book for so long delaying our notice of it. The only apology we can offer in extenuation, is, that we desired to read it carefully before expressing opinions concerning its merits, and have thus been obliged to delay longer than is our usual custom.

The work treats upon sexual precosity, masturbation in childhood, continence, celebicy, early marriages, early betrothals, long engagements, incontinence, masturbation in youth and adult, insanity arising from masturbation, virility, marriage, sexual intercourse in marriages, marital excesses, impotence, sexual indifference, infecundity, unfruitfulness, spermatorrhœa, and other topics too numerous to mention. The style is unexceptionably tasty, and the book is well worthy careful perusal. As a popular work, to be placed in the hands of young people of both sexes, it is sufficiently professional to make its teachings respected, while it is plain enough to be comprehended by all. Upon many subjects treated in this work even physicians are often times unsettled in their opinions, and the work so far as we are able to judge will confirm, in truthful and correct views. Some of the chapters are upon topics not strictly medical in themselves considered, yet they have a medical bearing, and it is well to understand the influence of marriage, early betrothal, long engagements, etc., etc., upon the habits and health of the young. The opinions of the author are sustained by numerous cases which forcibly illustrate the importance of these subjects to physicians.

